# Viewpoint oscillation improves the perception of distance travelled in static observers but not during treadmill walking

**DOI:** 10.1007/s00221-020-05786-y

**Published:** 2020-03-25

**Authors:** Martin Bossard, Cédric Goulon, Daniel Mestre

**Affiliations:** 1 Aix-Marseille Univ, CNRS, ISM, Marseille, France; 2 School of Psychology, Cardiff University, Cardiff, UK

**Keywords:** Optic flow, Distance travelled estimation, Self-motion, Treadmill walking, Viewpoint oscillations

## Abstract

Optic flow has been found to be a significant cue for static observers’ perception of distance travelled. In previous research conducted in a large-scale immersive display (CAVE), adding viewpoint oscillations to a radial optic flow simulating forward self-motion was found to modulate this perception. In the present two experiments, we investigated (1) whether the improved distance travelled perceptions observed with an oscillating viewpoint in a CAVE were also obtained when the subjects were wearing a head mounted display (HMD, an Oculus Rift) and (2) whether the absence of viewpoint oscillations during treadmill walking was liable to affect the subjects’ perception of self-motion. In Experiment 1, static observers performed a distance travelled estimation task while facing either a purely linear visual simulation of self-motion (in depth) or the same flow in addition to viewpoint oscillations based on the subjects’ own head oscillations previously recorded during treadmill walking. Results show that the benefits of viewpoint oscillations observed in a CAVE persisted when the participants were wearing an HMD. In Experiment 2, participants had to carry out the same task while walking on a treadmill under two different visual conditions simulating self-motion in depth: the one with and the other without the visual consequences of their head translations. Results showed that viewpoint oscillations did not improve the accuracy of subjects’ distance travelled estimations. A comparison between the two experiments showed that adding internal dynamic information about actual self-motion to visual information did not allow participants better estimates.

## Introduction

During locomotion, spatio-temporal information is delivered by the pattern of optic flow (dynamic visual information, Gibson 1950). Optic flow information is known to play a key role in many aspects of self-motion perception, including distance travelled (Campos et al. 2010; Frenz and Lappe 2005; Lappe et al. 2007; Redlick et al. 2001), speed of self-motion (Banton et al. 2005; Larish and Flach 1990), heading (Warren and Hannon 1988), and time-to-collision (Lee and Lishman 1975). By dissociating these cues from dynamic information of other kinds, many authors have focused specifically on static observers’ ability to use optic flow alone to perceive the distance travelled. It has been established, for example, that individuals are able to use the optic flow fairly accurately to discriminate and visually simulated distances travelled (Bremmer and Lappe 1999; Frenz and Lappe 2005; Redlick et al. 2001; Sun et al. 2004).

Other dynamic cues, including vestibular, proprioceptive, and motor efference signals, have also been found to contribute to performing various kinds of spatial behavior. Some authors have set up conditions under which subjects had no access to visual information and only inertial and proprioceptive cues were available during actual self-motion. The results of these studies clearly showed that humans are able to a previously seen distance accurately by walking the same distance, even when they are deprived of vision (Elliott 1986; Fukusima et al. 1997; Loomis et al. 1992; Mittelstaedt and Mittelstaedt 2001; Rieser et al. 1990; Sun et al. 2004; Thomson 1983).

All in all, the optic flow and other dynamic cues, therefore, definitely provide important information about self-motion. However, in the natural world, these two sources of information are redundant, which makes it difficult to determine exactly how each of them contributes to the perception of self-motion. For example, locomotion generates not only forward whole body self-movement but also smaller-scale “bob”, “sway”, and “lunge” head movements (Cutting et al. 1992; Hirasaki et al. 1999) triggering multiple sources of interdependent perceptual information. These oscillatory head movements are both linear and angular, and affect the visual scene in ways that can only be partially compensated for by eye movements (Grossman et al. 1989; von Grünau et al. 2007). In short, locomotion generates optic flow and other dynamic cues which are closely interrelated and difficult to dissociate.

Some studies have already shown the benefits to vection (the sensation of visually induced self-motion) of adding jittering or oscillating viewpoints to the visual forward self-motion in static observers. For example, contrary to the predictions of the sensory conflict theory, adding frontal-plane jitter or oscillations to an expanding optic flow pattern was reported to enhance the sensation of vection experienced by static observers by significantly increasing the duration of this sensation and decreasing its onset latency (Kim and Palmisano 2008; Nakamura 2013; Palmisano et al. 2000). The latter authors suggested several explanations for this effect. Kim and Palmisano (2008, 2010b) attributed this enhanced visual perception of self-motion to the fact that in stationary observers, viewpoint oscillations generate similar compensatory eye movements to those which normally occur during natural walking to stabilize the retinal image of the environment and stimulate the parietoinsular vestibular cortex, which plays a major role in vestibular sensory integration processes (Nishiike et al. 2002). In other studies (Kim and Palmisano 2010a; Palmisano and Kim 2009; Palmisano et al. 2012), these same authors suggested the hypothesis that jitter/oscillations of this kind may increase the global retinal motion and thus improve the sensation of self-motion.

On similar lines, we previously tested whether the perception of distance travelled was also affected by similar viewpoint oscillations to those induced by head motion during natural walking. In a preliminary series of experiments (Bossard et al. 2016), we observed that in comparison with a purely translational optic flow, additional viewpoint oscillations simulating the head oscillations that occur during natural walking (using a model presented by Lécuyer et al. 2006) improved the subjects’ estimation of the distance travelled in a large-scale immersive display (CAVE). The main conclusion reached on the basis of these experiments was that this effect may be due to an increase in the global retinal motion. However, the possible contribution of an “ecological” factor cannot be ruled out: a second series of experiments (Bossard and Mestre 2018) in which the viewpoint oscillation frequency was made to vary showed that the optimal performances were recorded in a similar range of frequencies to those of the head motion which occurs during natural walking.

The present study focused on the role of the dynamic information generated by locomotion, and by head movements in particular, during actual walking. The participants were walking on a treadmill, wearing an HMD, with which the optic flow information/inputs could be monitored. Given the interdependence between visual cues and other dynamic cues, the main question addressed here was whether the presence/absence of viewpoint oscillations during treadmill walking is liable to affect subjects’ perception of self-motion and hence, their perception of the distance travelled. Rather than addressing the relative contribution of viewpoint oscillations to the perception of visual self-motion, as done in previous studies, the present study involving the use of a treadmill enslaved to the visual scene to induce a more complete simulation of forward self-movement in terms of the sensory inputs received by the subjects. In addition, partly dissociating the visual consequences of head oscillations from the other dynamic cues generated by the subjects’ own locomotion yielded new insights into the contribution of each of these cues to the perception of self-motion.

In the first experiment, static observers had to carry out a distance travelled estimation task while facing either a purely linear visual simulation of self-motion (in depth) or one to which viewpoint oscillations directly based on their own head oscillations previously recorded during treadmill walking were added. Results confirm that the benefits of viewpoint oscillation are maintained in an HMD, as established as in our previous studies conducted in a CAVE system (Bossard et al. 2016; Bossard and Mestre 2018). The second experiment was designed to test whether the absence of viewpoint oscillations during treadmill walking (when visual consequences of the head translations generated by walking are deleted; non-oscillatory condition) affects subjects’ perception of self-motion in comparison with a more complete pattern of visual feedback other dynamic cues. The non-oscillatory condition, therefore, potentially created a sensory conflict between optic flow, vestibular and proprioceptive inputs. Results show that the benefits of viewpoint oscillation disappear when participants are walking on a treadmill, when other self-motion cues are available.

## Experiment one

### Method

#### Participants

Twenty participants (11 women and 9 men, mean age 24.6 ± 2.7 years, mean height 172.4 ± 8.3 cm) volunteered to take part in the experiment. They had no vestibular antecedents or disorders that might affect their locomotor performances. They all had normal or corrected-to-normal vision. All the participants gave their written informed consent prior to the experiment, in keeping with the 1964 Helsinki Declaration, and the study was approved by Aix-Marseille University’s Ethics Committee.

### Recording phase

#### Apparatus

This experiment was conducted using a treadmill (h/p Cosmos) setup available at the TechnoSport Technical Facility (https://technosport.univ-amu.fr/en). Participants wearing a head-mounted device (Oculus Rift) from the Mediterranean Virtual Reality Center (www.crvm.eu) were standing on the treadmill holding onto the treadmill bars, secured with a harness (for safety reasons). The Oculus Rift has several advantages over the traditional helmets used in the past. First, the field of view is about 110°, which is twice the size of that available in conventional systems, the spatial resolution is 1800 × 1200 pixels (for each eye) and the temporal resolution is 90 Hz. Secondly, inbuilt rate sensors record all changes in angular head orientation in three dimensions. Thirdly, it is packaged/equipped with a camera with which the user’s translations can be recorded. The latter two points make it possible to retransmit the visual scene with an imperceptible delay (Kim et al. 2015). This system is capable of displaying synchronous, spatially accurate stereoscopic views of virtual environments coupled, in real time, both with user’s behavior and also with the speed of the treadmill. This means that the scrolling of the visual scene is entirely driven by the scrolling of the treadmill. The ICE software (https://crvm.ism.univ-amu.fr/en/ice_3d_motor_virtual_reality.html), a proprietary software developed by the Institute of Movement Sciences (https://ism.univ-amu.fr/en), was used to build the environmental setup and control the experimental procedure.

### Procedure

**Fig. 1:**
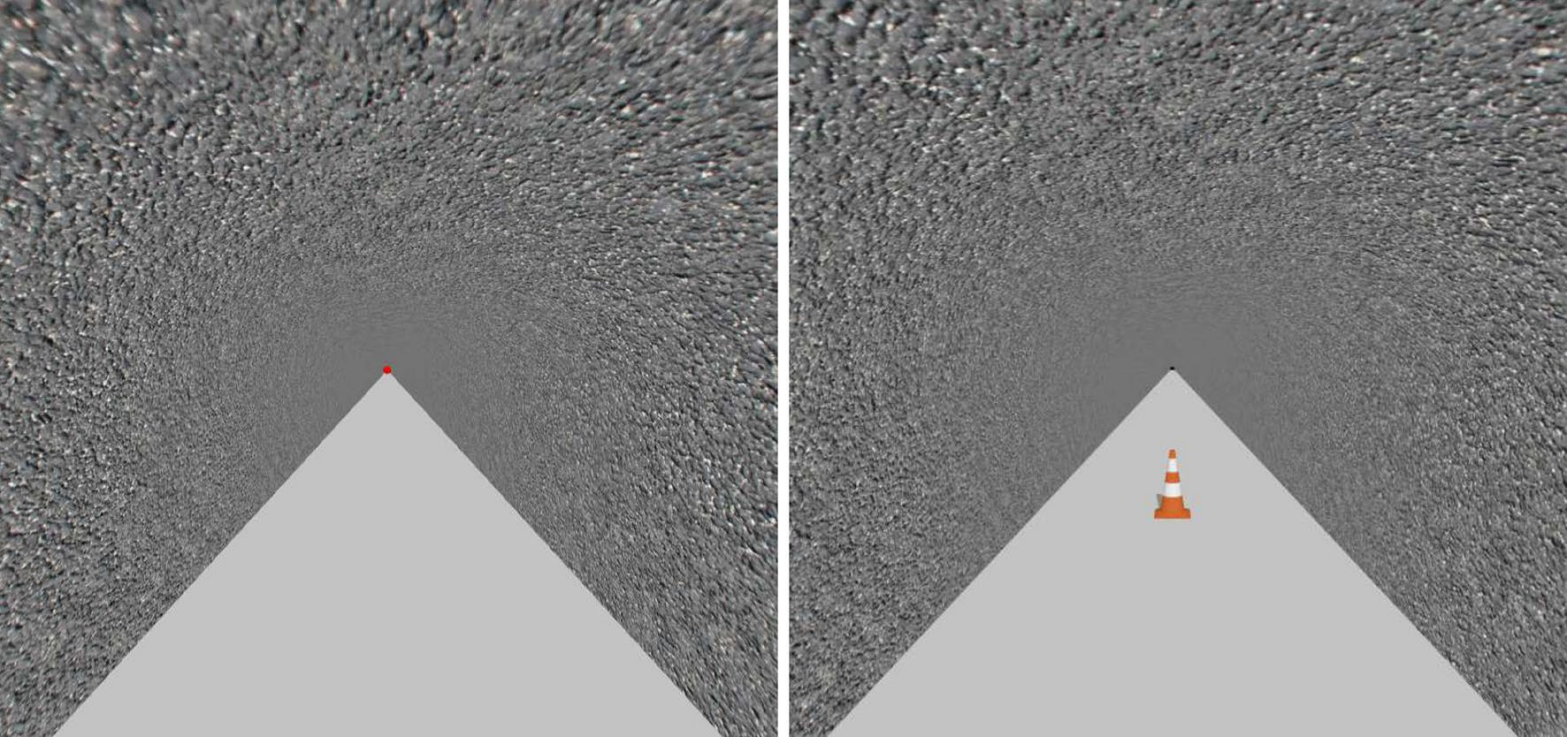
Visual scene in the recording phase (left) and the experimental phase (right)

Prior to the experimental phase, subjects underwent recordings of their own head movements while walking on the treadmill. These individual recordings were subsequently used in the experimental phase. The subjects were wearing a virtual reality helmet (Oculus Rift) in which they could see a tunnel, and headphones were used to present pink noise to mask the sound of the treadmill. This virtual tunnel was 3 m wide and the subject was placed in the center. The tunnel floor was graphically homogeneous and, therefore, devoid of all landmarks. A red and white spherical target could be seen in this tunnel, 20 m ahead at each individual subject’s eye-level. By clicking on the button of the Oculus Rift remote controller they were holding they could trigger both the movement of the visual scene and that of the servo-controlled treadmill. Subjects were instructed to fix/fixate the target (the relative position of which remained unchanged) visually while walking on the spot for 3 min. The speed of the treadmill and that of the visual scene increased gradually until reaching a set velocity of 1.2 m s^−1^ once the subject had covered a virtual distance of 3 m. Kinematic head data were recorded using the Oculus Rift camera (Fig. 1).

### Experimental phase

#### Apparatus

Stationary subjects wore a head-mounted display (Oculus Rift) in which an infinitely long straight tunnel was displayed (Fig. 1). No visual marks could be taken in the tunnel due to its nonsingular random texture. On the tunnel floor, the subject could see a target, a “roadworks” beacon of the usual size (height: 70 cm). This target was placed at initial virtual distances of 6, 12, 18, 24 or 30 m relative to the observer, depending on the trial (Bossard et al. 2016; Bossard and Mestre 2018; Plumert et al. 2005).

#### Procedure

The static subjects were placed in the middle of the virtual tunnel facing the target and were given the following instructions: (1) they would first hear a beep requesting them to estimate the distance to the target; (2) after a second beep, they could trigger the onset of the trial by pressing the remote control button, which had two simultaneous effects: the target would disappear and the virtual motion of the tunnel relative to the participant would be triggered.

While exposed to the optic flow stimulation, the participants, therefore, had to indicate when they thought they had reached the initial position of the target. When they felt they had reached this position, they had to click again on the remote control button. The second click would stop the motion of the tunnel, and after a period of two seconds, the onset of the following trial would be initiated. This same procedure was repeated in all the trials. Participants had to carry out 8 blocks of 10 trials (five distances [6, 12, 18, 24, and 30 m] × two optic flow conditions [Linear, and Oscillatory).

#### Conditions of virtual self-movement simulation: the optic flow factor

In the oscillatory condition, subjects were exposed to a visual scene inducing a sensation of self-motion based on their own walking pattern recorded during the previous recording phase. The virtual camera adopting the subject’s mobile viewpoint in the scenario was subjected to a translation, to which head oscillations on the anteroposterior, lateral, and vertical axes were added. The speed profile was the same for all the subjects: that imposed during the recording phase (increasing from 0 to 1.2 m s^−1^ during the first 3 m virtually travelled).

In the linear condition, the camera giving the subject’s viewpoint underwent a strictly linear translation, as if the camera were travelling on rails as on a movie set. The speed profile was exactly the same here as in the oscillatory condition.

#### Data analysis

Subjects’ estimates under the two optic flow simulation conditions were fitted using the leaky path integration model developed by Lappe et al. (2007). In this model, the subjects are assumed to monitor the current perceived distance *D*(*x*) to the target during the movement as a function of their simulated/virtual position (*x*), and to press the button when this distance becomes zero. The instantaneous change in *D* with respect to *x* is given by

$$\frac{{{\text{d}}D}}{{{\text{d}}x}} = - \;\alpha D - k$$

where *k* is the sensory gain (*k* = 1 in the case of an ideal observer) and *α* is the leaky integrator constant (*α* = 0 in the case of an ideal observer).

### Results and discussion

When they were exposed to scenes inducing the feeling of forward movement towards a previously seen distant target, subjects indicated that they had reached the target after travelling only 80% of the actual distance to the target on average. An ANOVA was conducted on the simulated distance travelled at the moment when the subjects responded (the dependent variable). This analysis involved three independent variables (Block_8_ × Distance_5_ × Optic Flow_2_). The results showed that the main effects involved were those of the block factor (*F* (7, 133) = 4.95, *p* < 0.001), the distance factor (*F* (4, 76) = 182.43, *p* < 0.001), and the optic flow factor (*F* (1, 19) = 22.87, *p* < 0.001). A significant interaction was also found to occur between the optic flow and distance factors (*F* (4, 76) = 3.5, *p* < 0.05).

**Fig. 2:**
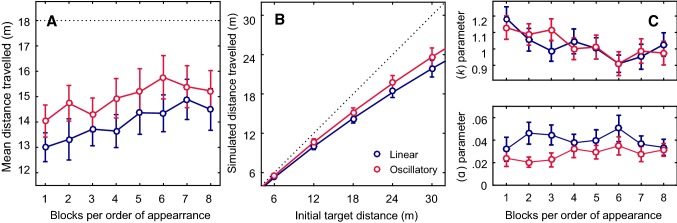
**a** Mean simulated distance travelled per block of trials. Data points are means based on 8 repetitions carried out by each of the 20 subjects, and error bars give the standard errors of the means. The dotted line gives the average initial target distance. **b** Distance travelled depending on the initial distance to the target in the linear (blue), and oscillatory (red) conditions. Data points are means based on 8 repetitions carried out by each of the 20 subjects, and error bars give the standard errors of the means. The dotted black line indicates the actual distances. Blue (*k* = 1.021; *α* = 0.04), and red (*k* = 1.027; *α* = 0.027) lines are the fits obtained by fitting the average data to the leaky integration model (Lappe et al. 2007). See the “Methods” section for details. **c** Mean sensory gain values (*k*) and leak parameter values (*α*) in the leaky spatial integrator model depending on the experimental block and optic flow conditions. Data points are means based on twenty subjects, and error bars give the standard errors of the means

The presence of a block factor effect indicates that the participants did not assess the distance travelled in the same way throughout the experiment. They tended to respond too early during the first few blocks, and their assessments tended to stabilize during the subsequent trials. As in our previous studies (Bossard et al. 2016; Bossard and Mestre 2018), further analyses were conducted on the last 4 blocks alone, to determine at what point the Block factor effect disappeared (*F* (3, 51) = 0.87, *p* > 0.05; see Fig. 2a).

The presence of a significant distance effect (*F* (4, 76) = 182.43, *p* < 0.001) indicates that the participants’ performances depended on the distance to be estimated. This finding suggests the existence of a positive correlation between the initial distance to the target and the subjects’ distance travelled estimates. Figure 2b shows the simulated distance travelled versus the distance to the initially seen static target under the two optic flow conditions (linear and oscillatory). In the case of the largest distances, subjects undershot the simulated distance travelled: when the initial target was 30 m away, for example, they responded after the simulated distance travelled was only 23 m on average, whereas with the shortest distances, they overshot the simulated distance travelled.

One of the main results obtained here was the presence of an optic flow condition effect (*F* (1, 19) = 22.87, *p* < 0.001). As can be seen from Fig. 2b, the simulated distance travelled in the oscillatory mode tended to match the real distances more closely than in the linear condition.

Figure 2b gives the simulated distance travelled depending on the initial distance to the target and the optic flow condition. The existence of a significant interaction between the latter two factors (*F* (4, 76) = 3.5, *p* < 0.05) means that the effect of the one factor varied depending on the modalities involved in the other factor. In other words, although an overall difference was observed between the responses produced, depending on the optic flow conditions, this does not mean that this was the case with all the distances tested. The results obtained in this study show that the oscillatory condition yielded more accurate distance travelled assessments than the linear conditions in the case of longer distances.

#### Leaky integrator model

All the subjects’ responses recorded in the two optic flow conditions and with the various initial target distances were fitted by the leaky spatial integrator model presented by Lappe et al. (2007) (see “Methods”) to determine the sensory gain (*k*) and the leak rate (*α*). An ANOVA was performed on each of these values (Block8 × Optic flow2). In the case of the gain parameter (*k*), the block factor was found to have significant effects (*F* (7, 133) = 5.32, *p* < 0.001; Fig. 2c), but not the optic flow condition (*F* (1, 19) = 0.06, *p* > 0.5). The second ANOVA showed on the contrary that the leak rate (*α*) did not differ between blocks (*F* (7, 133) = 0.92, *p* > 0.5), but that it differed between the optic flow conditions (*F* (1, 19) = 9.5, *p* < 0.01; Fig. 2c).

## Experiment two

Based on the use of an HMD, Experiment 1 showed the reproducibility of the results obtained in our previous studies (Bossard et al. 2016; Bossard and Mestre 2018) conducted in a CAVE system as regards the benefits to distance travelled perception of viewpoint oscillations in comparison with a purely linear simulation of self-motion. At this point, it was proposed to address the question as to whether the benefits of viewpoint oscillations are maintained during multisensory perception of self-motion or more specifically, whether or not the perception of distance travelled is affected when the visual consequences of head movements are removed from the visual simulation of forward self-motion during treadmill walking.

### Method

#### Participants

Thirty participants (14 women and 16 men, mean age 26.1 ± 6.8 years, mean height 172.4 ± 7.3 cm) volunteered to take part in the second experiment. They had no vestibular antecedents or disorders liable to affect their locomotor performances. They all had normal or corrected-to-normal vision. All the participants gave their written informed consent prior to the experiment, in keeping with the 1964 Helsinki Declaration, and the study was approved by Aix-Marseille University’s Ethics Committee.

#### Apparatus

The laboratory, the virtual reality device, and the virtual scene were all identical to those used in Experiment 1. The treadmill was the same as that used during the recording phase of Experiment 1.

#### Procedure

The procedure was practically identical to that used in Experiment 1, apart from the following difference. In addition to wearing a head-mounted virtual reality device (Oculus Rift) in which an infinitely long straight tunnel was displayed, subjects were standing on a treadmill to which the visual scene was enslaved (Fig. 1, right). Therefore, by starting to perform a trial, in addition to causing the disappearance of the target and setting the visual scene in motion, they also triggered the movement of the treadmill.

Apart from this difference, the task was exactly the same. Participants had to carry out 10 blocks of 10 trials (five distances [6, 12, 18, 24, and 30 m] × two optic flow conditions [non-oscillatory, and oscillatory]).

#### Conditions of virtual simulation of self-movement: the Optic flow factor

In the oscillatory condition, the virtual camera was enslaved by both the subject’s head movements and the treadmill velocity, which increased gradually from 0 to 1.2 m s^−1^ during the first 3 m travelled. In other words, to the visual environment, fixed relative to the Earth, was added a linear translation corresponding to the treadmill velocity.

In the non-oscillatory condition, the virtual camera was driven only by the treadmill velocity. The subject’s viewpoint obeyed a strictly linear translation at the same velocity here as in the first condition (at a velocity ranging from 0 to 1.2 m s^−1^ during the first 3 m) as if the camera was moving on straight horizontal rails, aligned with the tunnel, at the subjects’ eye level. In other words, the same linear translation as in the oscillatory condition was added to the visual environment, but here the visual environment was fixed relative to the participant’s head. This was achieved by deactivating the camera normally used to track the translational movements of the individual wearing the HMD.

### Results

#### Perceived distance travelled

The ANOVA involved three independent variables (Block_10_ × Distance_5_ × Optic flow_2_). The results show that the main effects involved here were the block factor (*F* (9, 261) = 4.82, *p* < 0.001), and the distance factor (*F* (4, 116) = 412.92, *p* < 0.001). A significant interaction was also found to occur between the block and distance factors (*F *(36, 1044) = 1.95, *p* < 0.001).

**Fig. 3:**
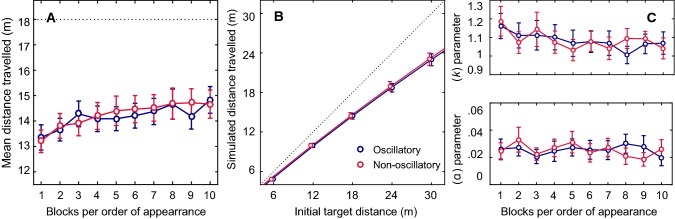
**a** Mean simulated distance travelled per block of trials. Data points are means based on 10 repetitions carried out by each of the 30 subjects, and error bars give the standard errors of the means. The dotted line gives the average initial target distance. **b** Distance travelled depending on the initial distance to the target in the oscillatory (blue) and non-oscillatory (red) conditions. Data points are means based on 10 repetitions carried out by each of the 30 subjects, and error bars give the standard errors of the means. The dotted black line indicates the actual distances. Blue and red lines are the fits obtained by fitting the average data to the leaky integration model. **c** Mean sensory gain values (*k*) and leak parameter values (*α*) in the leaky spatial integrator model depending on the experimental block and optic flow condition. Data points are means based on 30 subjects, and error bars give the standard errors of the means

The presence of a significant Block factor indicates that the participants did not assess the distance travelled in the same way throughout the experiment. They tended to respond too early during the first two blocks, and their assessments tended to stabilize during the subsequent trials (Fig. 3a). When the analysis was restricted to the last eight blocks, the effects of the Block factor disappeared (*F* (7, 203) = 1.89, *p* > 0.05).

As in the first experiment, the presence of a significant distance-related factor, *F* (4, 116) = 412.92, *p* < 0.001, indicates that the participants’ performance depended on the distance to be estimated. As in the first Experiment, the subjects undershot the virtual distance travelled more with large distances than with short distances to the target (Fig. 3a). When the initial target was 30 m away, for example, the subjects responded after the virtual distance travelled was only 23 m on average.

#### Leaky integrator model

All the subjects’ responses recorded in the two optic flow conditions and with the various initial target distances were fitted by the leaky spatial integrator model to compute the sensory gain (*k*) and the leak rate (*α*) parameters. The average *R*^2^ of these fits was 0.96 ± 0.04 in the oscillatory condition and 0.96 ± 0.08 in the non-oscillatory condition. An ANOVA was performed on each of these values (Block_10_ × Optic flow_2_). In the case of the gain parameter (*k*), as in the first experiment, the Block factor was found to have significant effects (*F* (9, 261) = 1.92, *p* < 0.05; Fig. 3c): the ANOVA showed the occurrence of a significant decrease in the value of parameter k, and this effect disappeared when the analysis was restricted to the last nine blocks: *F* (8, 232) = 0.86, *p* > 0.05. This analysis did not show the presence of any significant effect of the optic flow condition (*F* (1, 29) = 0.008, *p* > 0.5). A second ANOVA showed that the leak rate (*α*) did not differ between blocks (*F* (9, 261) = 0.73, *p* > 0.5) or between the optic flow conditions (*F* (1, 29) = 0, *p* > 0.5; Fig. 3c).

#### Head movements

In all the trials conducted during this experiment, the head movements’ spatial-frequency content was analyzed by calculating 2-D Fast Fourier Transforms (FFTs) on the data provided by the head-mounted display. An ANOVA was then conducted on the values of peaks corresponding to vertical head oscillations in all ten blocks, in the two optic flow conditions, and with the four longest distances, because the shortest distance did not always allow subjects to reach the stable speed (1.2 m s^−1^). This analysis, therefore, involved three independent variables (Block_10_ × Distance_4_ × Optic flow_2_). The results obtained here showed that these variables had no significant effects (Block: *F* (9, 171) = 1.00, *p* > 0.5; Distance: *F* (3, 57) = 1.75, *p* > 0.5; Optic Flow: *F* (1, 19) = 0.69, *p* > 0.5). In other words, participants’ vertical head oscillations, corresponding to their stepping frequency, did not differ between optic flow conditions (non-oscillatory condition: 1.7 ± 0.11 Hz; oscillatory condition: 1.71 ± 0.11 Hz).

#### Comparison between the two experiments

To compare results obtained in our two experiments, an ANOVA was performed on the simulated distance travelled in blocks number 5, 6, 7, and 8 in each experiment. Results obtained in these blocks are the only ones comparable, because they do not present a Block factor effect and participants were confronted to these blocks at the same moment in the two experiments. This analysis involved three independent variables Exp_2_ × (Block_4_ × Distance_5_ × Optic flow_2_) and showed the existence of a main effect of the Distance factor (*F* (4, 192) = 413.66, *p* < 0.001) and the Optic Flow factor (*F* (1, 48) = 9.17, *p* < 0.005). However, no significant interaction effects involving the Experiment factor was revealed by this analysis (Exp2 × Block4—*F* (3, 144) = 1, *p* > 0.05; Exp2 × Flux2—*F* (1, 48) = 3.9, *p* > 0.05; Exp2 × Distance5—*F* (4, 192) = 0.25, *p* > 0.05).

**Fig. 4:**
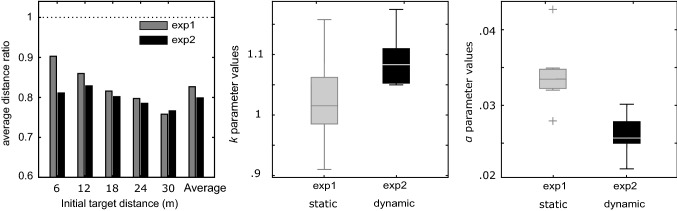
Comparison between average results obtained in Experiment 1 (static conditions) and Experiment 2 (dynamic conditions). **a** Average distance ratio between distance travelled estimates and the initial distance to the target. **b** Mean sensory gain values (*k*). **c** Mean leak parameter values (*α*) in the leaky spatial integrator model. Box plots are based on the values of the model’s parameters in Experiment 1 and 2 computed on the average of 8 and 10 blocks of trials carried out by 20 and 30 participants, respectively

However, subject assessments seem to maintain a more constant relationship with initial target distance in the second experiment (Fig. 4a). In other words, the error seems to be proportional to the initial target distance in Experiment 2, while it does not seem to be in Experiment 1. This impression is supported by a *t* test (independent samples) comparisons of the values of the leaky path integration model parameters, obtained by fitting the average data of each participant in both experiments (Fig. 4b, c). Indeed, while no difference was found concerning the k parameter (*t*(48) =  − 0.98, *p* > 0.05) between Experiment 1 and 2, values of α parameter were found to be higher in Experiment 1 in comparison to Experiment 2 (*t*(48) = 2.24, *p* < 0.01). The leaky integrator constant (*α*) is known to be responsible for the increase of the error with the increase of the initial target distance. Figure 4a illustrates this augmentation.

## General discussion

When they were exposed to scenes inducing a sensation of forward movement toward a previously seen distant target, subjects indicated that they had reached the target after traveling only 79.5% on average of the actual distance to the target (Experiment 1, 80%; and Experiment 2, 79%). As in previous studies in which the same procedure was used (Bossard et al. 2016; Bossard and Mestre 2018; Harris et al. 2012; Redlick et al. 2001), whether they were stationary or walking on a treadmill, subjects undershot the target in the case of large distances, but contrary to what was reported to occur in the latter studies, they also tended to undershoot it in the case of short distances.

All in all, these results are in agreement with those obtained in several studies on the perceptual compression of large distances in the real world on the basis of static visual cues (Loomis et al. 1992), as well as in virtual environments (Mohler et al. 2006; Thompson et al. 2003). This phenomenon was found to be generally more pronounced in virtual environments (Knapp and Loomis 2004; Loomis and Knapp 2003; Piryankova et al. 2013). However, the subjects’ underestimation of egocentric distances does not account for the systematically differential effects of our optic flow conditions in Experiment 1 on the subjects’ assessment of the distance travelled: regardless of their initial egocentric distance estimates, adding oscillatory viewpoint components was found to affect their perception of the distance travelled.

### Experiment 1

**Fig. 5:**
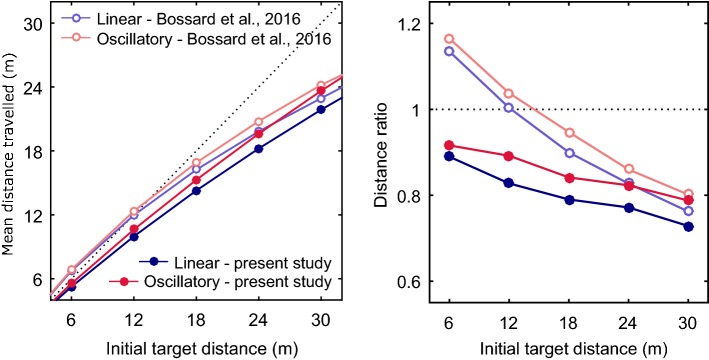
Comparison between the present results and those obtained by Bossard et al. (2016). Left, distance travelled depending on the initial distance to the target in linear and oscillatory (light blue and light red; Bossard et al. 2016), and in linear and oscillatory (dark blue and dark red; Experiment 1 in this study) conditions. Right, average distance ratio between distance travelled estimates and the initial distance to the target in the same four conditions

In Experiment 1, apart from the fact that the results obtained by Bossard et al. (2016) on the influence of viewpoint oscillations were confirmed with the use of an HMD, several differences stand out between the latter study and our own: as can be seen from Fig. 5, contrary to the findings made by Bossard et al. (2016), the present subjects did not overshoot the shorter distance to the target and seem to have been less influenced by the initial distance to the target. In Fig. 5, right; we have normalized participants’ assessments with respect to the initial target distance, resulting in a Distance ratio (Distance ratio = distance travelled estimates/initial distance to the target) which gives a better idea of the participants’ performances across initial distances. This study does not allow us to fully explain these results, because two parameters might be involved: (1) the egocentric distance might be perceived differently in an HMD from what occurred with the large-scale immersive display used by Bossard et al. (2016). For example, the distance to the screen has been found to be a factor influencing egocentric distance perception (Bruder et al. 2016), as is the field of view (Willemsen et al. 2009), which is wider in the CAVE than in the Oculus; or (2) the various speeds used might explain the differences between the results obtained: although Lappe et al. (2007) suggested that the integration process may be performed in space rather than time, Redlick et al. (2001) have shown that speed can influence distance travelled perceptions. Harris et al. (2012) have reported in particular that subjects tended to overestimate short distances more when the speed was 2 m s^−1^ rather than 1 m s^−1^. Further studies are, therefore, now required to determine the effects of these parameters.

In our previous study conducted in a CAVE environment (Bossard and Mestre 2018), we established that various oscillating viewpoint conditions do not generate pursuit eye movements in static observers. There is no reason why eye movement behavior might change in an HMD. It is, therefore, possible that the benefits of oscillating viewpoints to distance travelled perception may still be explained by the idea that viewpoint oscillation increases the global retinal motion and thus improves the sensation of self-motion.

### Experiment 2

During locomotion, it has been established that the eyes rotate in response to the head’s oscillations so as to maintain the gaze in space (Grossman et al. 1989; Hirasaki et al. 1999; Imai et al. 2001), according to a process called the Vestibulo-Ocular Reflex (VOR). The VOR is triggered by the activation of the vestibular system. The results obtained here regarding head movement generated during treadmill walking show that no difference was noticeable between the two visual conditions studied, while the VOR might have been expected to generate different retinal flows under these two visual conditions. In the oscillatory condition (the “natural” condition), the VOR is likely to stabilize the retinal flow (to attenuate the head’s oscillations) resulting in a “less” oscillatory situation; and the non-oscillatory condition, the VOR seems likely to generate more retinal flow oscillation than the oscillatory condition, resulting in more oscillations than in the oscillatory condition. According to the retinal motion hypothesis put forward in the case of static observers (Bossard and Mestre 2018; Palmisano et al. 2012), non-oscillatory conditions generating more retinal flow should predictably improve travelled distance perceptions, but this did not prove to be the case here.

In fact, while the absence of viewpoint oscillations during simulated forward motion was found to deteriorate path integration and distance travelled perception in static observers (Experiment 1), the distance travelled perception of participants walking on a treadmill (Experiment 2) were not significantly affected by the absence of viewpoint oscillations. No statistical differences were found between our two optic flow conditions, in terms of either the distance travelled estimates or the leaky path integrator model parameters. These results argue against an ecological explanation according to which a self-motion perception advantage might be expected to occur under oscillatory conditions—when visual, proprioceptive, efference copy, and vestibular (except for forward movement) information all match, while the visual information available under non-oscillatory conditions is in conflict with other dynamic information generated by body and head movements. The advantages of viewpoint oscillation seem to be annulled here by the presence of internal dynamic cues. These conclusions are supported by previous findings (Campos et al. 2012, 2014) indicating that the contribution of internal dynamic cues predominates over that of the dynamic visual flow in the estimation of distances travelled. The present results support the idea that proprioceptive/efference copy information contributes significantly to spatial processing in the absence of complete visual feedback inputs contributing to the perception of forward self-motion. These results also show that the absence of visual consequences of the head movements that occur during treadmill walking, creating a sensory conflict, does not degrade subjects’ distance travelled perceptions.

As Kim and Palmisano pointed out in (2010b), it is possible that non-oscillatory conditions trigger eye movements of another kind. In the latter study, these authors recorded observers’ eye movements, while they were performing translational head oscillations when presented with a radially expanding flow synchronized with their head movements, in either the ipsilateral (minimal sensory conflict) or a contralateral (high sensory conflict) direction. These authors reported that ocular following responses can differ in terms of their amplitude, depending on the direction in which the stimulation is applied. It might, therefore, be interesting in future experiments to measure eye movements under non-oscillatory conditions to see whether ocular following responses of this kind occur during locomotion.

### Comparisons between the two experiments

It has been observed that the presence (or absence) of internal dynamic information during distance travelled estimations did not seem to affect the subjects’ estimates. However, the mean value of the *α* parameter has been shown to be significantly lower in Experiment 2 compared to Experiment 1 (Fig. 4c) and it is known that the lower the value, the higher the correlation between subjects’ estimations and the initial position of the target. Figure 4a, in which the Distance ratio (described above) is presented, illustrates the consequences of the values of *α* parameter: subjects’ assessments seem to have maintained a more constant relationship with the initial target distance in Experiment 2 than in Experiment 1. In Experiment 2, only vestibular information about linear self-motion in depth and the fact that the participants were holding the stationary treadmill handbars gave an indication of stationarity, whereas in Experiment 1, no vestibular oscillations, and no proprioceptive or efference copy information about locomotion were present. In other words, when visual self-motion is simulated, the proprioceptive and vestibular systems cannot be "turned off", and so they constantly deliver information about the subjects’ stationarity, which is potentially deleterious to their self-motion perception. The larger leak rate average observed in Experiment 1 (under static conditions) than in Experiment 2 (Fig. 4c) suggests that over longer distances, responses produced under static conditions will deviate more from the ideal responses than those elicited under dynamic conditions. To sum up, these findings suggest that additional information about self-motion does not yield more accurate travelled distance estimations for the range of initial distances we explored but allow us to predict that for larger distance it might be the case.

Finally, these results cannot be explained in terms of visual speed perception during linear self-motion. As Durgin et al. (2005) have shown, the perceived speed of the optic flow decreases during treadmill walking (as well as during passive linear self-motion and normal walking). These findings should have led us to expect a less important under-shooting (or an over-shooting) of the target to occur in the second experiment (dynamic conditions) than in the first one (static conditions) in the present study. No clear-cut differences of this kind were found to exist, however, between the two experiments.

## Conclusions

These two experiments show that viewpoint oscillations influence static subjects’ distance travelled perceptions but not those of treadmill-walking subjects. The results of the first experiment showed that the advantage of viewpoint oscillations observed in static observers persists in participants wearing a head-mounted display (HMD) in comparison with large-scale immersive display (a CAVE) and when the speed of the visually simulated forward self-motion increases. In the second experiment, the benefits of viewpoint oscillation were found to disappear when the participants had to carry out the same task during treadmill walking. The sensory conflict or decorrelation between the visual and other dynamic information about the pattern of locomotion had no effect on the subjects’ distance travelled estimates. In other words, the absence of visual consequences of head motion (the non-stabilization of the world) did not affect the accuracy of the distance travelled estimates in treadmill walking subjects. The comparison of our two experiments showed that the presence (or not) of internal dynamic cues does not allow more accurate distance travelled estimates for the range of initial distances studied but the leaky integrator model allows us to predict that, for longer distance than 30 m, it might be the case.
